# Supplementary material for: Pyruvate Dehydrogenase Kinase 1 inhibition mediated oxidative phosphorylation enhancement in cartilage promotes osteoarthritis progression
Source: BMC Musculoskelet Disord. 2023 Jul 20;24:597. doi: 10.1186/s12891-023-06585-6 (PMC10357736; doi:10.1186/s12891-023-06585-6)
Supplement: Supplementary file 1 — Additional file 1. [file 12891_2023_6585_MOESM1_ESM.zip › BP.Gsea.1653623667859/GOBP_DEOXYRIBONUCLEOSIDE_MONOPHOSPHATE_METABOLIC_PROCESS.html]

Details for gene set GOBP\_DEOXYRIBONUCLEOSIDE\_MONOPHOSPHATE\_METABOLIC\_PROCESS[GSEA]

|  || Dataset | 55457\_collapsed\_to\_symbols.cls.cls #OA\_versus\_normal.cls.cls #OA\_versus\_normal\_repos |
| Phenotype | cls.cls#OA\_versus\_normal\_repos |
| Upregulated in class | OA |
| GeneSet | GOBP\_DEOXYRIBONUCLEOSIDE\_MONOPHOSPHATE\_METABOLIC\_PROCESS |
| Enrichment Score (ES) | 0.47444135 |
| Normalized Enrichment Score (NES) | 1.4909568 |
| Nominal p-value | 0.06030151 |
| FDR q-value | 0.3870345 |
| FWER p-Value | 1.0 |
Table: GSEA Results Summary

  

Fig 1: Enrichment plot: GOBP\_DEOXYRIBONUCLEOSIDE\_MONOPHOSPHATE\_METABOLIC\_PROCESS      
 Profile of the Running ES Score & Positions of GeneSet Members on the Rank Ordered List

  

| SYMBOL | TITLE | RANK IN GENE LIST | RANK METRIC SCORE | RUNNING ES | CORE ENRICHMENT || 1 | DGUOK | deoxyguanosine kinase [Source:HGNC Symbol;Acc:HGNC:2858] | 96 | 0.917 | 0.1187 | Yes |
| 2 | ADK | adenosine kinase [Source:HGNC Symbol;Acc:HGNC:257] | 127 | 0.878 | 0.2372 | Yes |
| 3 | DPYS | dihydropyrimidinase [Source:HGNC Symbol;Acc:HGNC:3013] | 509 | 0.656 | 0.2978 | Yes |
| 4 | DNPH1 | 2'-deoxynucleoside 5'-phosphate N-hydrolase 1 [Source:HGNC Symbol;Acc:HGNC:21218] | 719 | 0.589 | 0.3626 | Yes |
| 5 | DCK | deoxycytidine kinase [Source:HGNC Symbol;Acc:HGNC:2704] | 920 | 0.537 | 0.4209 | Yes |
| 6 | DCTD | dCMP deaminase [Source:HGNC Symbol;Acc:HGNC:2710] | 1107 | 0.494 | 0.4744 | Yes |
| 7 | NT5C | "5', 3'-nucleotidase, cytosolic [Source:HGNC Symbol;Acc:HGNC:17144]" | 1852 | 0.379 | 0.4688 | No |
| 8 | TK1 | thymidine kinase 1 [Source:HGNC Symbol;Acc:HGNC:11830] | 2909 | 0.259 | 0.4225 | No |
| 9 | TYMP | thymidine phosphorylase [Source:HGNC Symbol;Acc:HGNC:3148] | 3588 | 0.195 | 0.3967 | No |
| 10 | DPYD | dihydropyrimidine dehydrogenase [Source:HGNC Symbol;Acc:HGNC:3012] | 3830 | 0.173 | 0.4018 | No |
| 11 | TYMS | thymidylate synthetase [Source:HGNC Symbol;Acc:HGNC:12441] | 4328 | 0.126 | 0.3805 | No |
| 12 | DUT | deoxyuridine triphosphatase [Source:HGNC Symbol;Acc:HGNC:3078] | 5981 | -0.003 | 0.2527 | No |
| 13 | TK2 | thymidine kinase 2 [Source:HGNC Symbol;Acc:HGNC:11831] | 7468 | -0.116 | 0.1532 | No |
| 14 | NT5M | "5',3'-nucleotidase, mitochondrial [Source:HGNC Symbol;Acc:HGNC:15769]" | 7781 | -0.140 | 0.1483 | No |
| 15 | ADA | adenosine deaminase [Source:HGNC Symbol;Acc:HGNC:186] | 8105 | -0.163 | 0.1457 | No |
| 16 | XDH | xanthine dehydrogenase [Source:HGNC Symbol;Acc:HGNC:12805] | 8366 | -0.183 | 0.1507 | No |
| 17 | UPB1 | beta-ureidopropionase 1 [Source:HGNC Symbol;Acc:HGNC:16297] | 9609 | -0.284 | 0.0933 | No |
| 18 | PNP | purine nucleoside phosphorylase [Source:HGNC Symbol;Acc:HGNC:7892] | 9657 | -0.288 | 0.1292 | No |
| 19 | SHMT1 | serine hydroxymethyltransferase 1 [Source:HGNC Symbol;Acc:HGNC:10850] | 9727 | -0.294 | 0.1644 | No |
| 20 | UPP1 | uridine phosphorylase 1 [Source:HGNC Symbol;Acc:HGNC:12576] | 12013 | -0.595 | 0.0689 | No |
Table: GSEA details [plain text format]

  

Fig 2: GOBP\_DEOXYRIBONUCLEOSIDE\_MONOPHOSPHATE\_METABOLIC\_PROCESS      
 Blue-Pink O' Gram in the Space of the Analyzed GeneSet

  

Fig 3: GOBP\_DEOXYRIBONUCLEOSIDE\_MONOPHOSPHATE\_METABOLIC\_PROCESS: Random ES distribution      
 Gene set null distribution of ES for **GOBP\_DEOXYRIBONUCLEOSIDE\_MONOPHOSPHATE\_METABOLIC\_PROCESS**
